# A Novel Working Memory Task-Induced EEG Response (WM-TIER) Feature Extraction Framework for Detecting Alzheimer’s Disease and Mild Cognitive Impairment

**DOI:** 10.3390/bios15050289

**Published:** 2025-05-04

**Authors:** Yi-Hung Liu, Thanh-Tung Trinh, Chia-Fen Tsai, Jie-Kai Yang, Chun-Ying Lee, Chien-Te Wu

**Affiliations:** 1 Institute of Electrical and Control Engineering, National Yang Ming Chiao Tung University, Hsinchu 30010, Taiwan; yhliu182@nycu.edu.tw (Y.-H.L.); jackyang.ee12@nycu.edu.tw (J.-K.Y.); 2 Graduate Institute of Manufacturing Technology, College of Mechanical and Electrical Engineering, National Taipei University of Technology, Taipei 10608, Taiwan; tungtt@hsb.edu.vn (T.-T.T.); leech@ntut.edu.tw (C.-Y.L.); 3 Department of Information and Communication Technology, Hanoi School of Business and Management, Vietnam National University, Hanoi 100000, Vietnam; 4 Department of Psychiatry, Division of Geriatric Psychiatry, Taipei Veterans General Hospital, Taipei 11217, Taiwan; chiafentsai@gmail.com; 5 Faculty of Medicine, National Yang Ming Chiao Tung University, Taipei 11221, Taiwan; 6 Department of Mechanical Engineering, National Taipei University of Technology, Taipei 10608, Taiwan; 7 Department of Occupational Therapy, College of Public Health and Health Professions, University of Florida, Gainesville, FL 32608, USA; 8 Center for Cognitive Aging and Memory, McKnight Brain Institute, University of Florida, Gainesville, FL 32608, USA

**Keywords:** electroencephalography, Alzheimer’s disease, mild cognitive impairment, machine learning, working memory task-induced EEG response

## Abstract

The electroencephalography (EEG)-based approach provides a promising low-cost and non-invasive approach to the early detection of pathological cognitive decline. However, current studies predominantly utilize EEGs from resting state (rsEEG) or task-state (task EEG), posing challenges to classification performances due to the unconstrainted nature of mind wandering during resting state or the inherent inter-participant variability from task execution. To address these limitations, this study proposes a novel feature extraction framework, working memory task-induced EEG response (WM-TIER), which adjusts task EEG features by rsEEG features and leverages the often-overlooked inter-state changes of EEGs. We recorded EEGs from 21 AD individuals, 24 MCI individuals, and 27 healthy controls (HC) during both resting and working memory task conditions. We then compared the classification performance of WM-TIER to the conventional rsEEG or task EEG framework. For each framework, three feature types were examined: relative power, spectral coherence, and filter-bank phase lag index (FB-PLI). Our results indicated that FB-PLI-based WM-TIER features provide (1) better AD/MCI versus HC classification accuracy than rsEEG and task EEG frameworks and (2) high accuracy for three-class classification of AD vs. MCI vs. HC. These findings suggest that the EEG-based rest-to-task state transition can be an effective neural marker for the early detection of pathological cognitive decline.

## 1. Introduction

Beyond prevention, early intervention for individuals with early signs of cognitive decline is crucial for reducing disease burdens caused by dementia [1,2,3,4]. Recent research has strived to identify the earliest stage of cognitive impairment, offering a therapeutic window for early and non-pharmacological interventions [4,5,6,7]. Mild cognitive impairment (MCI) represents an intermediate stage between healthy aging and dementia, where individuals exhibit cognitive deficits that do not significantly impact their ability to perform daily activities [8,9,10,11]. Identifying reliable predictive markers for the early detection of MCI is essential for implementing timely and effective interventions [4].

Although several biomarkers have been used to aid in the identification of individuals with MCI and Alzheimer’s disease (AD), these methods are often expensive and require specialized equipment or invasive procedures [8,9,11], such as positron emission tomography (PET), cerebrospinal fluid (CSF), and magnetic resonance imaging (MRI). As a low-cost, non-invasive alternative, EEGs have emerged as a promising approach for the early detection of cognitive decline. Specifically, resting state EEGs (rsEEG) collected while participants are at rest without any specific task engagement are widely used due to their low burden to participants, especially older people [12]. Previously, rsEEG has shown promise in the binary classification of AD [13,14,15,16,17,18,19,20,21,22,23,24,25,26,27,28,29,30]/MCI [26,27,31,32,33,34,35,36,37,38] vs. HC. However, the classification performance tested is still limited. Further improving the binary classification accuracy has been considered a critical issue to be addressed.

Despite the undisputed advantages of rsEEGs in clinical research, several limitations should be noted. First, rsEEGs, by definition, are recorded with no task instructions and leave participants’ minds unguided and “wandering” between various thoughts [39,40]. Such heterogeneity of underlying mental activities during “resting” poses challenges to the test-retest reliability of the extracted EEG markers for classification [41,42]. Second, the variability of mental states immediately before the rsEEG recording would introduce different influences on brain dynamics during the following resting state. For example, significant changes in resting state alpha power were observed between rsEEG before and after performing verbal recognition tasks [43]. Third, a long recording period without tasks (especially with eyes closed rsEEG) could lead to drowsiness that reduces the quality of the acquired data and/or increases inter-participant variability.

To overcome these limitations, EEGs collected while participants were performing tasks (task EEG) provide a complementary alternative for extracting classification effective EEG markers, primarily because task EEGs offer more information regarding the neural substrates engaged in specific mental tasks [44]. In the realm of AD/MCI vs. HC classification, working memory-related tasks are frequently adopted as a behavioral probe to reliably show memory dysfunction in individuals with AD/MCI [45,46,47,48]. Commonly adopted subtypes of working memory tasks include auditory recognition tasks [49,50,51,52], backward counting tasks [53], visual detection tasks [54,55,56], and delayed match-to-sample tasks [57]. Despite the limited literature on the subject, working memory task EEG has reportedly demonstrated better performances than rsEEG in AD/MCI versus HC classification [53,58].

Although task-based EEG (task EEG) shows promising improvements in classification performance, the lack of adequate correction for the corresponding “baseline” state for each individual can introduce significant non-pathological inter-participant variability. This inherent variability in EEG markers obtained during tasks poses additional challenges to classification accuracy. To address this issue, the neurophysiological changes from resting state to task implementation state (the inter-state transition) may provide more valuable information for detecting early indicators that distinguish between cognitive decline groups and HC groups [44,59,60]. For example, an EEG-based study indicated that AD/MCI groups show increased functional integration in the cognitive state compared to the resting state [44]. Also, the functional MRI-based study by Wang et al. [60] observed that while the functional connectivity between the bilateral precuneus and the right inferior parietal lobule increases from rest to a working memory state in HC participants, no significant change occurs between these states in individuals with MCI. In summary, the previous AD/MCI vs. HC EEG classification studies using either rsEEG or task EEG alone may overlook valuable information found in the inter-state changes of EEG dynamics.

Extracting a set of distinguishing neural signatures from EEG dynamics is crucial to the development of an accurate machine learning method for MCI/AD classification. Unlike most previous studies that primarily focused on analyzing existing EEG features in either resting or task states, the current study aims to design a new feature extraction framework to enhance the classification performance of existing EEG features. To achieve this goal, we propose a novel framework to extract the response to changes from the resting state to a working memory task state, called the working memory task-induced EEG response (WM-TIER) feature extraction framework.

Specifically, the framework was applied to examine the difference of functional connectivity alteration from resting state to task state between the AD/MCI group and HC group. The current study focuses on the phase lag index (PLI)-based functional connectivity [61,62], which is less sensitive to the volume conduction effect present in traditional coherence-based connectivity measures [63] and was recently demonstrated to show potential in AD detection [64,65]. Its performance was compared to other commonly adopted measures, including coherence and the relative power between an electrode pair, which also showed promising results in our previous study related to MCI detection [32]. Furthermore, according to our previous results focusing on the spectral power feature, changes in the cortical electrical activity of different frequency bands are associated with cognitive impairment in various degrees. It is expected that integrating the functional connectivity features from different frequency bands would increase the sensitivity in detecting MCI and/or AD. Accordingly, based on the WM-TIER feature extraction framework, the current study further proposed a filter-bank PLI (FB-PLI) WM-TIER feature, which extracts the PLI features from multi-channel EEG signals of different bands, calculates the inter-state changes of these features, and then reduces the feature dimension through a machine learning-based optimal feature subset selection strategy.

In recent years, traditional machine learning algorithms, including K-nearest neighbors (KNNs), linear discriminant analysis (LDA), and support vector machines (SVM)s, have been widely applied in EEG-based classification for neurodegenerative conditions. These methods are popular due to their simplicity, interpretability, and efficiency in handling relatively small EEG datasets. Reasonable performance in EEG-based detection of MCI or AD has been reported with KNN ([12,49,50,66,67,68] for MCI, [12,18,19,20,22,23,66,69] for AD), LDA ([26,70,71,72,73] for MCI, [16,26,70,71] for AD), and SVM ([33,52,53,74,75,76,77,78] for MCI, [15,21,53,69,76,77,78,79,80,81,82] for AD). However, most existing studies apply these classifiers with EEG features extracted from a single brain state (either rest or task), limiting their ability to capture the dynamic neural alterations associated with cognitive decline. To bridge this gap, our study leverages these classical classifiers in the newly proposed feature extraction framework (WM-TIER) that captures the transitional changes from the rest to task states, aiming to enhance the classification accuracy in AD/MCI detection.

The main contributions of the presented study are summarized as follows:

(1) To the best of our knowledge, the proposed WM-TIER feature extraction architecture is novel and has not been proposed before. It offers two main advantages: (1) it reduces the influence of the inter-individual variability, and (2) it leverages the often-overlooked information embedded in the inter-state changes of EEG dynamics.
(2) Given that the FB-PLI WM-TIER features encapsulate information on EEG connectivity across different frequency bands and the dynamics of EEG changes from rest to task and are further optimized using a machine learning-based feature selection strategy, we hypothesize that these features will achieve more effective classification between AD/MCI and HC compared to either rsEEG or task EEG PLI features. This hypothesis will be substantiated by our results in Section 3.1.
(3) Additionally, we investigated the differences in FB-PLI connectivity features extracted under the WM-TIER framework (i.e., the FB-PLI WM-TIER features) between the three groups: HC, MCI, and AD (Section 3.2 and Section 3.3). This approach allows an in-depth examination of PLI variations within the same group across different states and highlights the differences in state transitions among the different groups. The results of this analysis provide interpretable neural markers that could be used as references for auxiliary assessments in future applications. Furthermore, these markers hold the potential to be used as objective indicators for evaluating treatment outcomes should real clinical applications arise in the future.

## 2. Materials and Methods

### 2.1. Participants

The present study included both old data from a subgroup reported in a previous publication [78] and new data. Specifically, four participants with Alzheimer’s disease (AD) from the previous study [78] were removed because three participants did not complete the memory tasks and one participant was too old. Additionally, data from two new participants were added. At the end, 21 participants with AD (8 females, mean age of 71.19 ± 5.04 y/o), 24 participants with mild cognitive impairment (MCI) (14 females, mean age of 70.96 ± 8.2 y/o), and 27 healthy control (HC) participants (17 females, mean age of 69.93 ± 4.98 y/o) were included (Table 1). The collection of EEG data was conducted from July 2017 to July 2020 at an outpatient Memory Clinic of Taipei Veterans General Hospital, a first-class medical center (~3000 beds). Diagnosis of AD or MCI in the study group was validated at clinical consensus meetings by board-qualified psychiatrists based on the National Institute on Aging and the Alzheimer’s Association’s (NIA-AA) core clinical criteria [83,84] and the outcomes of clinical interviews, neuropsychological exams, laboratory results, and imaging techniques (CT and/or MRI). Individuals in the control group were recruited through advertisements and verified as not presenting any of the NIA-AA criteria for all-cause dementia. Additionally, all healthy control individuals were assessed with the Montreal Cognitive Assessment (MoCA) [85] and showed normal scores adjusted for education years (>24) [86].

For all three groups, the exclusion criteria were (1) current major psychiatric comorbidity (clinically diagnosed within 6 months prior to the neuropsychological evaluation), (2) sensory and/or motor deficits that may confound the cognitive function evaluation, and (3) neurological illness or condition that may interfere with cognitive performance. The study protocol was reviewed and approved by the institutional review board of Taipei Veterans General Hospital (IRB No: 2017-06-009A). Written informed consents were collected before participating in the study from all participants or their legally authorized guardian according to the Declaration of Helsinki.

### 2.2. Task Procedures

**Figure 1:**
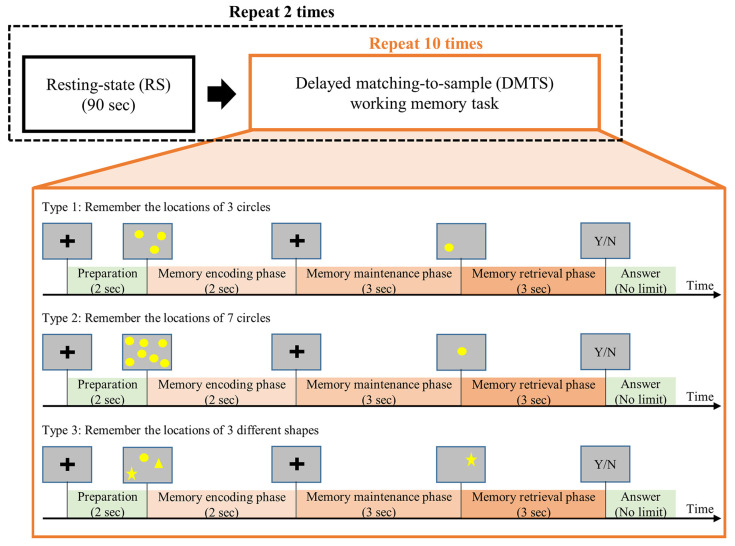
The task procedures. Each participant underwent two sessions of resting state conditions and two sessions of delayed matching-to-sample (DMTS) tasks (3 types). Type 1 and Type 2 tasks require participants to remember the positions of stimuli, and the Type 3 task requires participants to remember both the shapes and positions of stimuli.

In the current study, all participants underwent two sessions of resting state (RS) condition and two sessions of working memory (WM) condition with the following order: RS-WM-RS-WM (Figure 1). In the RS condition, participants were instructed to keep their eyes open and gently fixate on a centrally displayed cross without moving or deliberately thinking anything on purpose. Each RS session lasts 90 s. In the WM condition, participants performed three types of delayed matching-to-sample (DMTS) tasks [87,88] with 10 trials for each type.

Each DMTS trial was structured with three phases. First, a set of to-be-remembered sample stimuli was displayed on the screen during the memory-encoding (ME) phase (2 s). The stimuli were then removed from the screen during the memory-maintaining (MM) phase (3 s), and participants were instructed to maintain the information in their working memory. Next, a question stimulus was displayed on the screen for 3 s during the memory-retrieval (MR) phase, and participants were asked to judge whether the contents of the question stimulus matched those in the to-be-remembered sample stimuli. After the MR phase, participants answered the question with a button press without time constraint.

The three types of DMTS tasks differed in terms of to-be-remembered contents. In Type 1, participants were asked to remember the positions of three circles. In Type 2, participants were asked to remember the positions of seven circles. In Type 3, participants were asked to remember the positions of three different shapes (a circle, a square, and a star). For all three types, the positions for the stimuli were randomized across different trials.

### 2.3. EEG Data Acquisition and Preprocessing

EEG signals were recorded with a 33-channel electro-cap connected to a 40-channel NuAmps amplifier (Compumedics USA, Charlotte, NC, USA). The layout of the Ag/AgCl electrodes followed the international 10–20 system, covering frontal (FP1, FP2, F3, F4, F7, F8, and Fz), central (FC3, FC4, FCz, C3, C4, and Cz), parietal (CP3, CP4, CPz, P3, P4, and Pz), occipital (O1, O2, and Oz), left temporal (FT7, T3, TP7, and T5), and right temporal (FT8, T4, TP8, and T6). The impedance between the electrodes and skin was kept below 10 K Ohm by applying electrogels. The ground electrode was at the forehead and also used as the physical reference electrode during recording. The EEG data were then digitally re-referenced online to the average of the potentials of the mastoid electrodes A1 and A2: Fz = Fz − (A1 + A2)/2. The remaining 30 channels were utilized to record EEG data for analysis. Electrodes attached to the right side of the right eye and above the left eye were used to monitor electrooculography (EOG) signals. EEG and EOG signals were amplified, band-pass filtered (0.5–100 Hz), and transformed to digital signals with a sampling frequency of 500 Hz. The EOG artifacts were removed from the EEG signals using the artifact removal software tool Scan 4.5 provided by the NeuroScan. Moreover, contamination from other possible artifact components, such as generic discontinuities and electromyography (EMG) signals, was further removed by using the independent component analysis (ICA, Infomax, EEGLAB [89]) and the ADJUST algorithm [90].

### 2.4. FB-PLI WM-TIER Feature Extraction

In the current study, only the first session of the resting state EEGs (rsEEGs) was used for further analysis because the second session of the resting state EEGs may be affected by the first session of the working memory task. The 90 s resting state EEG signals of the first session were segmented into 45 non-overlapped epochs of 2 s length, and then 20 epochs among the 45 were randomly selected for analysis. As for the task EEG, we only analyzed the two phases of ME and MM. Accordingly, 20 EEG epochs of 2 s length in the ME phase and 20 EEG epochs of 2 s length in the MM phase for each type (Type 1/Type 2/Type 3) were analyzed. Note that although the MM phase lasted for 3 s, only the first 2 s of the recorded EEG data were used for analysis. We excluded EEG data from the MR phase in the current analyses due to the presence of significant artifacts during this phase, particularly in participants with AD. In the MR phase, participants were required to make a memory recognition judgment within a 3 s window, which involved not only memory retrieval processes but also motor-preparation-related activities (e.g., subtle muscle tension, finger or hand preparation for upcoming button press), facial micro-expressions, and eye blinks—especially under time pressure. These issues were particularly pronounced in older participants with severe cognitive impairments, who often exhibited greater variability in response preparation. During the data collection in the current study, we observed that AD participants often tend to show noticeable limb and head movements even before the 3 s MR phase had ended. In contrast, such patterns were rarely observed in participants in either the HC or MCI group. Moreover, during signal processing, visual inspection of data also indicated that there were signals with extremely large amplitudes and high frequencies in the raw MR-phase EEG data from most AD participants. Such electromyography (EMG)-related artifacts caused by muscle activity were difficult to remove completely even with artifact removal procedures. Therefore, the current study did not conduct the analysis on the EEGs from the MR phase.

#### 2.4.1. Stage 1: FB-PLI Feature Extraction

We filtered an EEG signal into five different frequency bands, including delta (1–4 Hz), theta (4–8 Hz), alpha (8–13 Hz), beta (13–30 Hz), and gamma (30–45 Hz) bands. An EEG time series x(t) is band-pass filtered, and then the corresponding analytic signal a(t), a complex-valued function, is represented as:

(1) $$a\left(t\right)=x\left(t\right)+i\overset{˘}{x}\left(t\right)$$

where $\overset{˘}{x}\left(t\right)$ is the Hilbert transform of x(t),

(2) $$\overset{˘}{x}\left(t\right)=\frac{1}{\pi }p.v.\int_{-\infty }^{+\infty }\frac{x\left(t\right)}{t-\tau }d\tau$$

where p.v. refers to the Cauchy principal value. The instantaneous phase of the signal can then be obtained:

(3) $$\emptyset \left(t\right)=\arctan\frac{\overset{˘}{x}\left(t\right)}{x\left(t\right)}$$

When two EEG signals from two different electrodes are functionally connected, the phase difference of their ongoing electrical activity over time will be consistent, whereas when they are not connected, the phase differences will be random. Thus, PLI measures the asymmetry of the phase difference (∆∅) distribution between two signals around zero. The phase difference was calculated by subtracting the phase of one signal from the phase of the other signal at each time point. A PLI value ranges from 0 to 1, where a value of 0 indicates no phase locking or locking with zero <modulus π>, and a value of 1 indicates perfect phase locking [61]:

(4) $$\mathrm{PLI}=\left|\frac{1}{L}\sum_{k=0}^{L-1}\mathrm{sign}\left[\sin\left(∆\emptyset \left(t_{k}\right)\right)\right]\right|$$

here, $t_{k}$ are discrete time steps, L is the number of samples, sign stands for the signum function, | | and indicates the absolute value.

PLI was calculated for each epoch. Thus, for each epoch, a total of 2175 PLI features ($5\;bands\times \left(30\times 29/2\right)\;electrode\;pairs$) were extracted for resting state and each type of working-memory task, respectively. The PLI features of resting state were further averaged over epochs. The PLI features of the working-memory task were first averaged across types and then averaged across the epochs. As a result, the 2175 PLI features from different frequency bands and different electrode pairs were extracted from a participant’s EEG signals, which constitute an FB-PLI feature vector. In other words, three vectors were obtained, including the vectors of dimension D from resting state, ME phase, and MM phase, respectively, where D=2175 in this case of FB-PLI. Notice that the latter two are the ones calculated from the DMTS based working memory task’s EEGs.

In the current study, we also compared FB-PLI with other types of commonly used features, including RP and coherence, introduced in the following.

##### Relative Power (RP)

Unlike the conventional single electrode-based relative power [36,91], e.g., the ratio of alpha power to beta power, here, we applied the inter-electrode relative power, which demonstrates the asymmetry in EEG spectral power between two electrodes and has shown better performance than the spectral band powers of single electrodes in both MCI and major depressive disorder detection in our previous works [33,92]. First, the band power (BP) of each band was first calculated by the Fast Fourier Transform (FFT) for each EEG epoch and then averaged over all epochs. The RP between a pair of electrodes *X* and *Y* at a specific band was subsequently calculated by:

(5) $$\mathrm{RP}=\frac{P(X)-P(Y)}{P(X)+P(Y)}$$

where P is the ratio of the power of the band of interest to the total power of the entire frequency range of 1–45 Hz. Since there were 5 bands and 30 electrodes, for each participant, a total of 2175 RP features for resting state and for each type of working-memory task were extracted, respectively. The RP features of the working-memory task were further averaged over types.

##### Coherence (Coh)

The coherency $C_{\mathrm{xy}}\left(f\right)$ between two EEG signals x(t) and y(t) at frequency f, obtained from electrodes *X*, *Y*, respectively, is a similarity-based measure in the frequency domain between the two electrodes [93]. Here, we use the magnitude-squared coherence:

(6) $${Coh(f)=\left|C_{\mathrm{xy}}\left(f\right)\right|}^{2}=\frac{{\left|G_{\mathrm{xy}}\left(f\right)\right|}^{2}}{G_{\mathrm{xx}}\left(f\right)G_{\mathrm{yy}}\left(f\right)}$$

where $G_{\mathrm{xx}}\left(f\right)$ and $G_{\mathrm{yy}}\left(f\right)$ are auto-spectra of x(t) and y(t) on band f, respectively, and $G_{\mathrm{xy}}\left(f\right)$ is the cross-spectra across the frequency band f between the signals x(t) and y(t). Note that the auto-spectra and cross-spectra are estimated from the spectrum of each EEG epoch:

(7) $$G_{\mathrm{xx}}\left(f\right)=\frac{1}{n_{e}}\sum_{e=1}^{n_{e}}{\left|X_{e}(f)\right|}^{2}$$

(8) $$G_{\mathrm{yy}}\left(f\right)=\frac{1}{n_{e}}\sum_{e=1}^{n_{e}}{\left|Y_{e}(f)\right|}^{2}$$

(9) $$G_{\mathrm{xy}}\left(f\right)=\frac{1}{n_{e}}\sum_{e=1}^{n_{e}}X_{e}(f)Y_{e}^{*}(f)$$

where $n_{e}$ is the number of EEG epochs, $X_{e}(f)$ and $Y_{e}(f)$ are the complex FFTs of the *e*th epoch of x(t) and y(t), respectively.

The coherence value ranges between 0 (two signals are uncorrelated) and 1 (two signals are identical). With 5 bands and 30 electrodes, a total of 2175 coherence features were extracted from resting state and each type of working-memory task, respectively. The coherence features of ME and MM tasks were further averaged across types, respectively.

#### 2.4.2. Stage 2: Task-Related Response Calculation and Feature Concatenation

**Figure 2:**
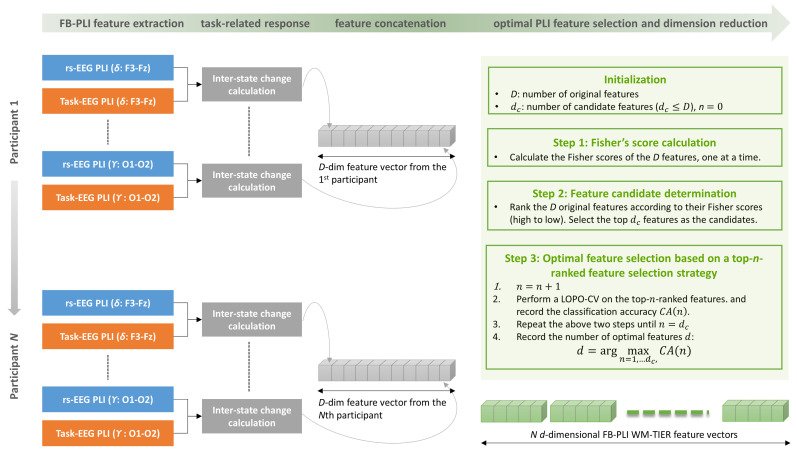
An example of the FB-PLI WM-TIER feature extraction and performance evaluation procedure. The task EEG includes features extracted from either the memory encoding (ME) or memory maintenance (MM) phase. When ME EEG is used, the extracted feature is a *d*-dimensional FB-PLI ME-TIER feature vector.

The working memory task-induced EEG response with the FB-PLI feature (i.e., the FB-PLI WM-TIER) and its extraction procedure is illustrated in Figure 2. In stage 2, we aim to capture the EEG responses induced by ME and MM tasks, respectively. The response should be capable of capturing the percentage change of the EEG dynamics from the resting state to the working memory task. To this end, supposing that $f_{i}^{rs}$ and $f_{i}^{task}$ are the *i*th FB-PLI features for the resting state and the working memory task, respectively, i=1,…, D, the inter-state change for the *i*th feature is given by

(10) $$\mathrm{inter\_state}\;\mathrm{change}=\frac{f_{i}^{task}-f_{i}^{rs}}{f_{i}^{rs}}$$

The inter-state change values for the features are then concatenated to a vector of D dimension (Figure 2).

#### 2.4.3. Stage 3: Optimal PLI Feature Selection and Dimension Reduction

The number of features (*D* = 2175) is considerably larger compared to the number of participants. Overly high feature numbers not only cause a high computational complexity but also increase the risk of model overfitting. This stage aims at selecting the optimal subset among the 2175 PLI features by applying Fisher’s criterion and a top-*n*-ranked feature selection strategy. Feature selection can be achieved by embedded, wrapper, or filter approaches. Different from the first two, the filter approach is independent of the classifier and computationally cheaper. Thus, it can be easily implemented as a fast-screening tool for selecting a set of candidate features among a large number of features. Fisher’s criterion [94] is a popular method in this category. We adopted this method to calculate the F-score for each PLI feature in the D-dimensional vector to reduce the feature dimension. The F-score of a feature presents the ratio of the between-class variation to the within-class variation in a feature space. A higher F-score indicates a larger separation between classes in a given feature space. By contrast, a noisier feature has a lower F-score. We ranked the 2175 features according to their F-scores in a descending order and then selected the top $d_{c}$ features as the candidates ($d_{c}\ll 2175$).

We further determined the optimal feature subset among the $d_{c}$ candidates by the top-n-ranked feature selection strategy suggested in [95]. Following this strategy, we assessed the classification accuracy using the top-n-ranked features. For example, n=2 indicates that both top-1 and top-2 features are used. The classification accuracy was assessed by performing leave-one-participant-out cross-validation (LOPO-CV) on the dataset with *N* data points, where a data point is an n-dimensional vector. LOPO-CV is an objective method for evaluating the participant-independent classification performance of a chosen classifier [96]. In every fold of the LOPO-CV, data points from the N−1 participants were used to train the chosen classifier, and then the n-dimensional data point from the remaining participant was used for testing. This step was performed repeatedly until every participant’s data point had served as the test data point once. The classification accuracy is the number of correctly classified participants divided by *N*. Here, the LOPO-CV was conducted once for every n ($n=1,2,\ldots ,\;d_{c}$). Finally, the optimal features were the ones giving the highest LOPO-CV classification accuracy. The above procedure is summarized in Figure 2. Let d be the number of the optimal features. Finally, the top-d optimal features constitute an FB-PLI WM-TIER feature vector.

### 2.5. Classifiers and Classification Tasks

#### 2.5.1. Classifiers

Since the current study focuses on the design of a novel feature extraction framework, we only applied three types of commonly used classifiers to assess the LOPO accuracy, including K-nearest neighbor (*K*NN), linear discriminant analysis (LDA), and support vector machine (SVM). The three chosen machine learning classifiers are based on different learning principles that simply try to find optimal decision boundaries, without further feature extraction from the to-be-examined resting-state, task-state, and the proposed WM-TIER features. Therefore, they are suitable for accessing the distinctive performances of these features. *K*NN (we set K=3 in this study) can be used to solve a binary or a multi-class classification, and both LDA and SVM are binary classifiers.

LDA finds a linear decision boundary in the space of data points to separate two classes. For an unseen data x, its predicted label is calculated by the decision function:

(11) $$D_{\mathrm{LDA}}\left(x\right)={\left(\mu_{1}-\mu_{2}\right)}^{t}\Sigma^{-1}x-\frac{1}{2}{\left(\mu_{1}-\mu_{2}\right)}^{t}\Sigma^{-1}\left(\mu_{1}+\mu_{2}\right)-\ln\left(\frac{w_{1}p_{2}}{w_{2}p_{1}}\right)$$

where t denotes the transpose of a matrix, $\mu_{1}$ and $\mu_{2}$ are the mean vectors of the training data of positive and negative classes, respectively, Σ is the covariance matrix of the training data, $w_{1}$ and $w_{2}$ are the penalty weights for positive and negative classes, respectively, and $p_{1}$ and $p_{2}$ are the *a priori* probabilities of positive and negative classes, respectively. Here, we set $w_{1}=w_{2}$. The test data point x is classified as positive if $D_{\mathrm{LDA}}\left(x\right)>\;0$; otherwise, it belongs to the negative class.

SVM maps the training data $x_{i\;},\;i=1,\ldots .,\;N,$ into a higher-dimensional feature space F via a nonlinear mapping φ. In this transformed space, SVM finds an optimal separating hyperplane $w^{T}\varphi \left(x\right)+b=0$ that maximizes the margin between classes and minimizes the training error. The decision function for SVM is

(12) $$D_{SVM}\left(x\right)=\sum_{x_{i\;}\in SV}\alpha_{i}y_{i}k\left(x_{i,\;},x\right)+b$$

where $0\leq \alpha_{i}\leq C$ are Lagrange multipliers, $y_{i}\in [-1,+1]$ are the class labels for the training data, and C is a penalty parameter. The term SV denotes the set of support vectors—the training data points satisfying $0<\alpha_{i}\leq C$. The bias of the hyperplane b can be obtained through the Kuhn–Tucker condition and $k\left(\;,\right)$ represents a kernel function, which computes the inner product of two mapped data points. Here, we used the Gaussian kernel $k\left(x_{i,\;},x\right)=exp\left(-\gamma {\left\|x_{i}-x\right\|}^{2}\right),$ where γ is the kernel parameter. The class label of an unseen data x is predicted as positive if $D_{\mathrm{SVM}}\left(x\right)\;>\;0$; otherwise, x is classified as the negative class.

#### 2.5.2. Classification

In the current study, we were interested in analyzing the optimal features for the AD-HC, MCI-H, and AD-HC classifications, respectively. Therefore, we performed the feature selection and dimension reduction on each of the three binary classifications. For each binary classification, the aforementioned LOPO-CV and top-*n*-ranked feature selection strategy were performed to determine the best feature subset, and the number of participants N was the sum of participants from two groups of interest.

We also analyzed the classification performance among different feature types. Here we organize the terms of the feature types as follows to facilitate a clear comparison:

(1) Task EEG features (i.e., ME-EEG and MM-EEG features) refer to those directly extracted from either the ME or MM phases without calculating the inter-state changes.
(2) Furthermore, task EEG features of RP, Coh, and FB-PLI were separately optimized by employing the top-*n*-feature ranked feature selection strategy combined with the LOPO-CV (i.e., the stage 3 in Figure 2).
(3) The features “TIER” are the features that went through the three stages shown in Figure 2. For example, ME-TIER of the RP feature is also a *d*-dimensional feature vector obtained by the WM-TIER feature extraction framework. Moreover, in the case of FB-PLI, both ME-TIER and MM-TIER are FB-PLI WM-TIER features.

In this study, we also address the 3-class classification (AD vs. MCI vs. HC) problem. Here we used the one-against-one (OAO) method and a majority voting strategy to extend the two binary classifiers LDA and SVM to solve the three-class classification problem. This approach involves training three LDA/SVM classifiers for AD-MCI, AD-HC, and MCI-HC classifications, respectively. Take the LDA classifier as an example. In the case of top-*n*-Fisher-score-ranked features, the LOPO-CV was conducted on the *N* data points from the *N* participants of HC, MCI, and AD, where a data point is an *n*-dimensional vector. In each fold of the LOPO-CV, one participant’s data point was used as the test data, and the remaining N−1 data points were reorganized into three different training sets for training three binary classifiers, where each training set was composed of the data points from two different groups. Then, the test data point was fed into the three classifiers, respectively, and then the three models generated three predicted labels. The final decision was made by the majority voting strategy.

## 3. Results

### 3.1. Comparision of Classification Accuracy Obtained from rsEEG, Task EEG, and WM-TIER Using Different Types of Features

**Figure 3:**
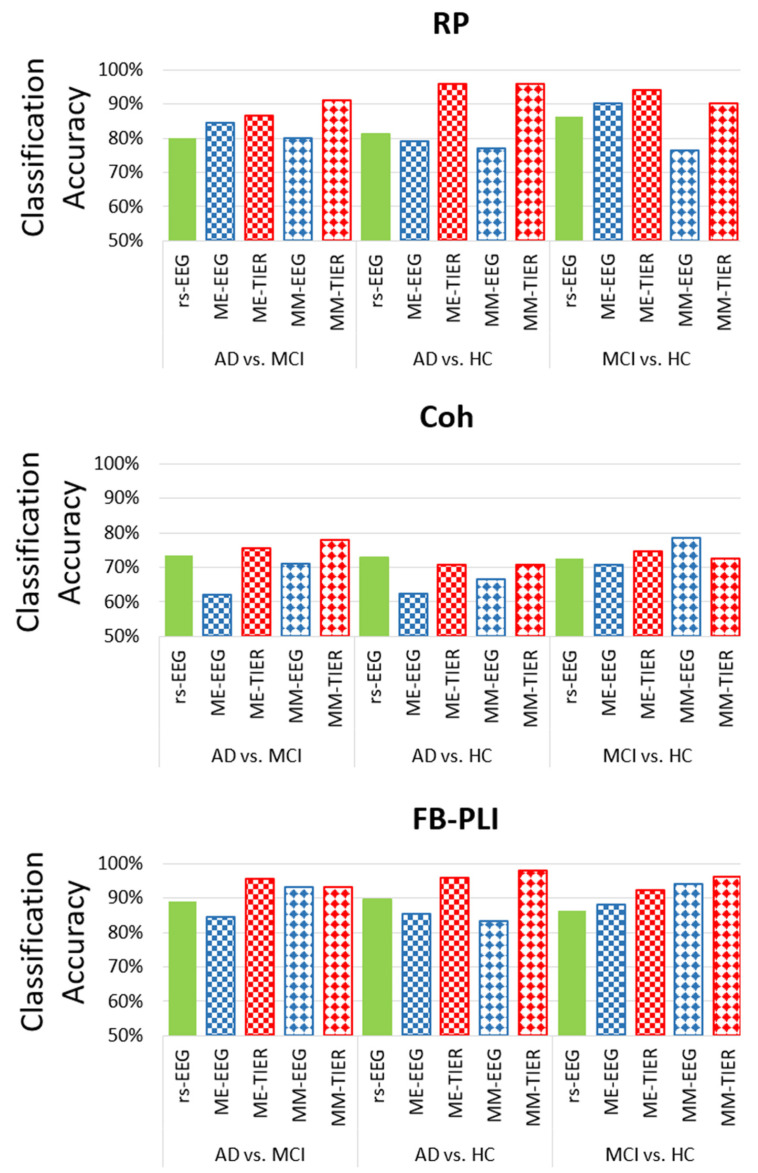
The highest LOPO-CV classification accuracy achieved from resting state EEG, working memory task EEG (ME-EEG and MM-EEG), and working memory task-induced EEG response (ME-TIER and MM-TIER) in different features (RP, Coh, and PLI) with LDA classifier.

Figure 3 illustrates the maximum LOPO-CV classification accuracy given by different feature types extracted from different frameworks (i.e., rsEEG, task EEG, and WM-TIER) for the three binary classifications with the linear classifier LDA: AD vs. MCI, AD vs. HC, and MCI vs. HC. The results show that task EEG features did not consistently outperform rsEEG features (Figure 3). For example, in the classification of MCI vs. HC, the accuracy of ME-EEG RP (90.20%) was higher than that of rsEEG RP (86.30%), whereas the accuracy of ME-EEG Coh (70.62%) was slightly lower than that of rsEEG Coh (72.50%). This suggests that working memory task EEG is not necessarily more sensitive for MCI vs. HC classification than the commonly used rsEEG. However, the performances of WM-TIER features generally surpassed those of both rsEEG and task EEG features across different feature types (RP, Coh, or FB-PLI) and classification tasks. These patterns demonstrate the efficacy of using inter-state changes of selected features (RP, Coh, FB-PLI) as neural markers to improve classification accuracy.

Among all feature types, Coh exhibited the lowest classification performance, with its highest accuracies across all classification tasks below 80%: AD vs. MCI (77.80% by MM-TIER), AD vs. HC (70.80% by ME-TIER)), and MCI vs. HC (78.40% by MM-EEG). Conversely, across all combinations of feature types, the FB-PLI WM-TIER feature demonstrates the best classification accuracy for all classification tasks: 95.60% (ME-TIER) for AD vs. MCI, 97.92% (MM-TIER) for AD vs. HC, and 96.10% (MM-TIER) for MCI vs. HC. The results show that the proposed FB-PLI WM-TIER features not only outperform other feature types such as Coh and RP but also greatly enhance classification performance compared to PLI features extracted from rsEEG or task EEG alone.

**Figure 4:**
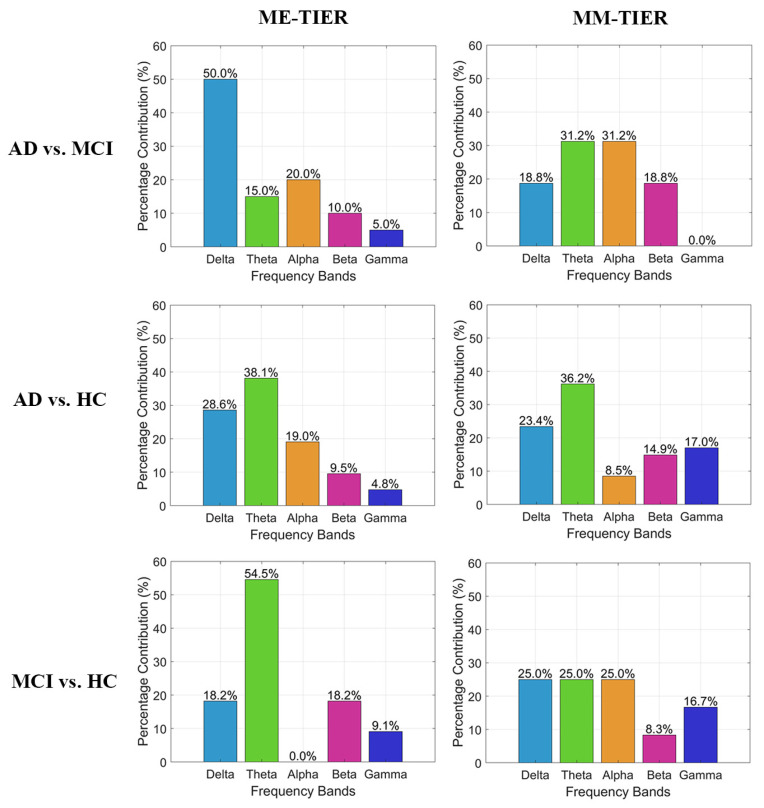
Band contribution of the optimal FB-PLI WM-TIER features for the three binary classifications.

Given that FB-PLI demonstrates the highest accuracy among the three feature types, we further analyze the characteristics of the optimal PLI feature subset within the WM-TIER feature extraction architecture. Figure 4 illustrates the proportion of each frequency band that contributes to the optimal WM-TIER features. Among the five bands, most contribution stems from slow oscillation, where the theta bands contribute to the majority across different task conditions (ME and MM) and classification types. This analysis also revealed that integrating functional connectivity features from different frequency bands is critical for achieving satisfactory classification between individuals with different cognitive impairment severities.

**Figure 5:**
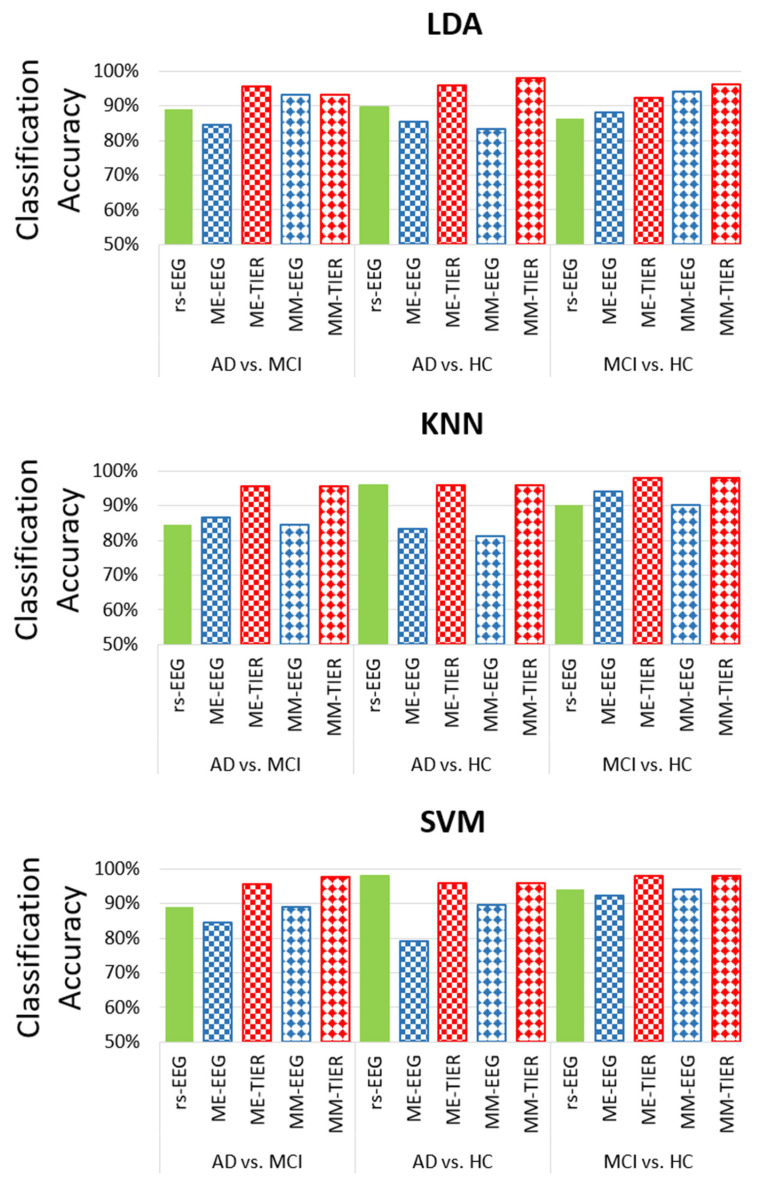
Comparison of the LOPO-CV classification accuracy across different classifiers and conditions (rsEEG, task EEG, and WM-TIER) using FB-PLI features.

We further compare the classification performance of the FB-PLI WM-TIER features across different classifiers. For the SVM classifier, we set C=100 and γ=1/(d*Var(X)), in which *d* is the number of features and Var(**X**) is the mean variance of all features in the input dataset **X**. As illustrated in Figure 5, both types of WM-TIER features (ME and MM) outperform both rsEEG and task EEG features in the MCI vs. HC and MCI vs. AD classification. Notably, however, rsEEG performs better in the AD vs. HC classification than task EEG or WM-TIER for the SVM classifier.

Finally, we tested the performance of FB-PLI WM-TIER features in a 3-class classification scenario (AD vs. MCI vs. HC) using different classifiers (LDA, KNN, SVM, LR). Here, another commonly used classifier—logistic regression (LR)—was also employed. In LR, two parameters, penalty (the norm used for regularization) and C (the inverse of regularization strength), were optimized by using a LOPO-CV procedure combined with grid search [97]. The search space included combinations of penalty ∈ {L1, L2} and C ∈ {0.001, 0.01, 0.1, 1, 10, 100}. To provide a more comprehensive comparison, we integrate the two-class classification results and the three-class classification results into one single table (Table 2). Our results showed that both types of WM-TIER features (ME and MM) consistently outperform both rsEEG and task EEG features. The highest 3-class classification accuracy of 93.06% was achieved by the combination of the LR classifier and the FB-PLI MM-TIER features, which is higher than the chance-level accuracy of 33.3% for a 3-class classification. Moreover, the results suggest that changes in FB-PLI-based connectivity from rest to both memory encoding and memory maintenance tasks play a critical role in classifying the severity of cognitive impairment.

**Figure 6:**
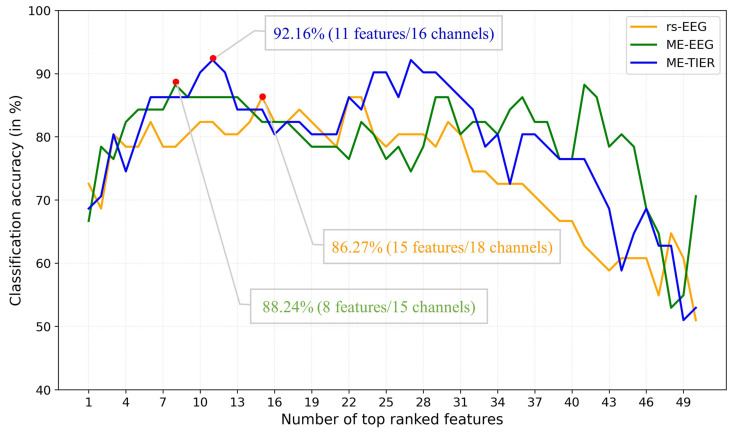
Comparison of MCI vs. HC classification accuracy using FB-PLI features between rsEEG, ME-EEG, and ME-TIER frameworks. The number of optimal features (and channels) is determined by applying the top-n-ranked feature selection strategy based on Fisher score and LOPO-CV.

In addition to classification accuracy, the number of optimal features d and the corresponding channels are also important metrics for evaluating the performance and practical applicability of the proposed model. Our approach demonstrates high classification accuracy while significantly reducing the number of features compared to the original 2175 features. It is noticed that the number of optimal features may vary with the feature type (rsEEG, task EEG, and WM-TIER), classifier chosen, and the classification task (AD-HC, MCI-HC, and AD-MCI). Taking the LDA classification of MCI vs. HC using FB-PLI features as an example, rsEEG achieved the highest LOPO-CV classification accuracy of 86.27% with 15 features derived from 18 channels, ME-EEG achieved the best accuracy of 88.24% with 8 features (15 channels), and MM-TIER further boosted the accuracy to 92.16% with 11 features (16 channels) (Figure 6). Table 2 shows that WM-TIER generally exhibits a significant superiority over both rsEEG and task EEG, and the optimal numbers of features are all much smaller than the original number of 2175 features.

### 3.2. The Inter-State Change of Theta Band PLI-Based Connectivity Within the AD, MCI, and HC Groups

**Figure 7:**
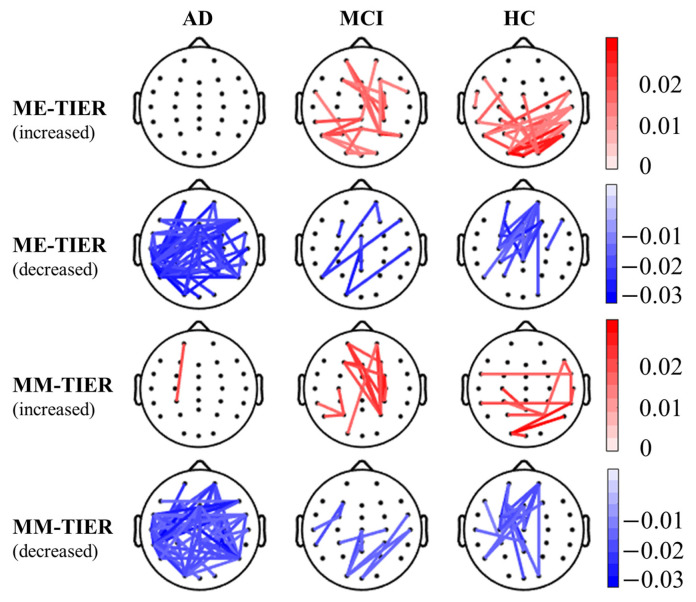
The theta-band PLI-based connectivity of WM-TIER (i.e., rest-to-task connectivity change) in the AD, MCI, and HC groups. Red lines indicate statistically significant connectivity increases during the task state compared to the resting state, whereas blue lines indicate statistically significant connectivity decreases during the task state compared to the resting state. The color bars indicate the strength of the corresponding connectivity increase or decrease.

Since the majority of effective features were found in the theta band (Figure 4), we further examined the topographical distribution of significant theta band PLI-based connectivity change induced by working memory tasks. Figure 7 illustrated significant changes in the theta band PLI-based functional connectivity from resting state to memory-encoding (ME) or memory-maintaining (MM) state for each group (corrected *p* < 0.05; details, please see Appendix A). Individuals with AD showed a dispersed pattern of connectivity decrease, with almost no connectivity increase. In contrast, individuals in both the MCI and HC groups showed region-specific connectivity increases and decreases induced by working memory tasks. In particular, the HC group showed a focalized connectivity increase over the parietal scalp region and a focalized connectivity decrease over the fronto-central scalp region. While the functional connectivity in AD patients decreases at almost all cortical regions, it shows an increase at occipital, parietal, and right temporal regions, along with a decrease at frontal and central regions in HC individuals. For MCI patients, the inter-state change of functional connectivity mostly occurs around the central area.

### 3.3. The Between-Group Difference of Theta Band PLI-Based Connectivity in rsEEG, Task EEG, and WM-TIER

**Figure 8:**
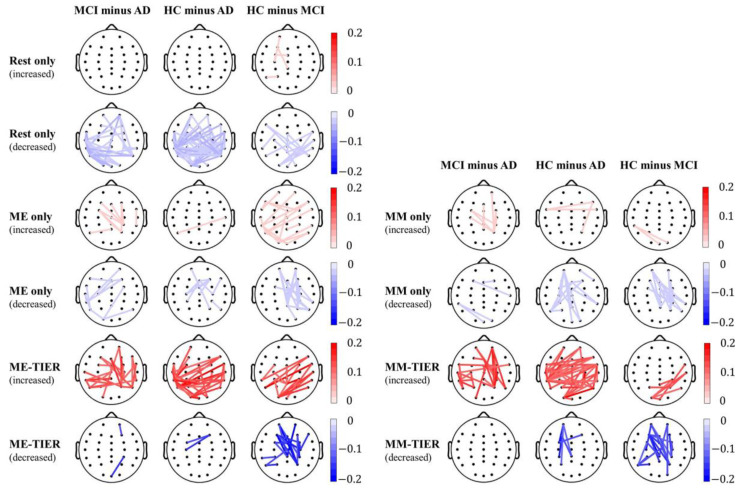
Group differences of theta band PLI-based connectivity in rsEEG, task EEG, and WM-TIER for the MCI minus AD, HC minus AD, and HC minus MCI contrasts. Red lines indicate statistically significant connectivity increases in the first group compared to the second group, whereas blue lines indicate statistically significant connectivity decreases in the first group compared to the second group. The color bars indicate the strength of the corresponding connectivity increase or decrease.

We further examined the difference of theta band PLI-based connectivity between groups in the rsEEG, task EEG, and WM-TIER to gain deeper insight into their topographical characteristics (Figure 8) (corrected *p* < 0.05; for details, please see Appendix B). In the rsEEG condition, the HC group showed a significant reduction in connectivity over the bilateral temporo-parietal scalp region as compared to both the AD and MCI groups. The AD group, representing a more severe form of dementia, showed an increase in theta band connectivity compared to both the MCI and HC groups.

In contrast, in the WM-TIER condition, the HC group demonstrated a significant increase in connectivity over the bilateral temporo-parietal scalp region as compared to both the AD and MCI groups. Additionally, a reduction in theta band connectivity over the fronto-central scalp region was observed in the HC minus MCI contrast, which was not seen in the HC minus AD contrast. Notably, the WM-TIER condition showed greater magnitudes of group differences, aligning with its better classification performance compared to the rsEEG or task EEG conditions. In the task EEG condition, a similar reduction in connectivity over the fronto-central scalp region was observed in the HC minus MCI contrast, albeit with a weaker magnitude.

## 4. Discussion

Most EEG-based AD/MCI detection systems use features extracted from EEG recorded during either the resting state or cognitive tasks, thereby missing the information of the inter-state EEG changes from resting to task. While recent research has explored the inter-state alterations in AD-related brain networks, such studies remain limited. To our best knowledge, no study has employed the inter-state EEG change feature as neural markers to build a classification model. In this study, we proposed a novel feature extraction framework to derive features from the working memory task-induced EEG response (WM-TIER) and compared its classification performance to the conventional frameworks of using features extracted from only rsEEG or task EEG. We examined three widely used EEG features in each framework: inter-electrode relative power, spectral coherence-based connectivity, and FB-PLI-based connectivity. The main results are twofold: (1) while features extracted from rsEEG and task EEG were fairly effective in classification performance; however, the WM-TIER framework provided superior classification performance, particularly in the HC vs. MCI and HC vs. AD classification. (2) FB-PLI-based WM-TIER achieved the highest classification performance.

### 4.1. The Effectiveness of rsEEG, Task EEG, and WM-TIER in Classification Between Groups

Existing literature reports inconsistent results regarding the potential for classification performance between features extracted from rsEEG vs. task EEG. Some studies suggest that cognitive task EEGs [44,53,98] or perceptual task EEGs [99] have an advantage over rsEEG, while others showed the opposite [98,100]. In the present study, we also observed such inconsistency: rsEEGs performed better in AD vs. HC classification, whereas task EEGs performed better in MCI vs. HC classification. One possible explanation is that individuals with AD cannot fully engage in memory tasks due to their more severe cognitive decline, introducing unstable disturbances to the recorded task EEG signals, which leads to lower classification performance. On the other hand, individuals with MCI may only show significant differences from the HC group during tasks, resulting in better performance on task EEG.

In contrast, the superior overall classification performance of WM-TIER compared to rsEEG or task EEG alone suggests that the state change of EEG features from resting state to cognitive task state provides more sensitive information on the differences between groups. This finding aligns with previous studies indicating that brains behave differently among patients and healthy individuals when they switch their cognitive state from rest to task [44,59,60]. It is important to note that the concept behind the task-induced EEG response of WM-TIER is different from event-related potentials (ERPs). To capture the EEG features “induced” by the task under the WM-TIER framework, we reference task EEGs to EEGs recorded during a resting state period before engaging in a series of working memory tasks, thus capturing a state change in a longer time scale (e.g., minutes). In this context, the neurophysiological signals of the resting state are unlikely to be influenced by subsequent cognitive tasks. In ERP, however, the responses are extracted with an epoch-wise baseline correction, aiming to capture a state change within a much shorter time scale (e.g., several hundred milliseconds). This process involves repetitive cycles of rest and task, which could lead to greater instability in the resting state baselines due to varying degrees of accumulated fatigue.

**Figure 9:**
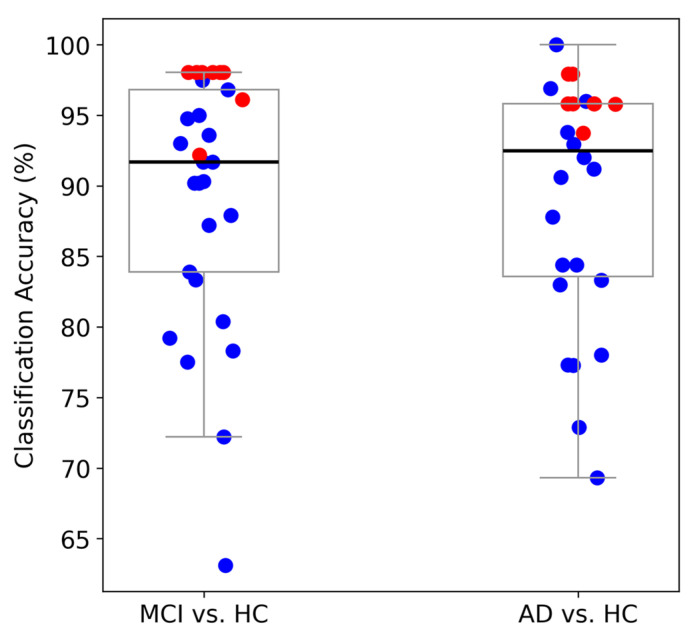
Comparison of binary classification performance based on LOPO-CV. Blue dots represent results from other approaches in previous studies, and red dots represent results from our study (red dots). Each boxplot includes eight red dots, which represent the classification accuracies from our proposed FB-PLI WM-TIER approach. For the MCI vs. HC task, the highest accuracies are 92.16% (ME-TIER with LDA), 98.04% (ME-TIER with KNN), 98.04% (ME-TIER with SVM), 98.04% (ME-TIER with LR), 96.08% (MM-TIER with LDA), 98.04% (MM-TIER with KNN), 98.04% (MM-TIER with SVM), and 98.04% (MM-TIER with LR). For the AD vs. HC task, the highest accuracies are 95.83% (ME-TIER with LDA), 95.83% (ME-TIER with KNN), 95.83% (ME-TIER with SVM), 93.75% (ME-TIER with LR), 97.92% (MM-TIER with LDA), 95.83% (MM-TIER with KNN), 95.83% (MM-TIER with SVM), and 97.92% (MM-TIER with LR) [see Table 2 for more details].

The 90–98.04% two-class classification performance with the FB-PLI-based WM-TIER approach provides a promising new direction compared to previous approaches. Compared to previous results with other feature extraction approaches and a similar LOPO-CV validation, the classification accuracy achieved by the proposed WM-TIER is among the highest (Figure 9). Although recent studies have reported EEG-based classification accuracies of up to 99% for AD/MCI vs. HC [18], these studies have several known limitations. These include the requirement for a sophisticated nonlinear approach [101], inflated performance due to the use of data from the same participants in both training and testing phases [35], or working with a relatively small dataset [76]. Furthermore, the proposed WM-TIER approach achieved a LOPO-CV accuracy of 91.67% for the challenging 3-class AD vs. MCI vs. HC classification using FB-PLI WM-TIER features and an SVM classifier. This performance appears to outperform the similar 3-class classification performance of 62.7~86.9% in other studies [26,34,35,37,76,101,102]. Although some studies have reported higher 3-class AD vs. MCI vs. HC classification accuracy than ours, these results should be taken with caution due to the data leakage problem [103] or the relatively small dataset [53,77]. Future studies will be needed to further investigate whether the WM-TIER approach can be used to establish a one-class model that delineates the severity gradient of pathological cognitive decline deviating from normal aging.

### 4.2. Increase of Theta Band Functional Connectivity During Resting State Due to Cognitive Decline

In the case of resting state condition, our results align with several EEG-based studies reporting increased theta band PLI-based functional connectivity in individuals with AD/MCI compared to HC [104,105]. This increase in theta band functional connectivity could be explained as a compensatory mechanism in the brains of those with AD/MCI [106]. The mechanism might involve alterations in brain behavior, such as neural reorganization or the forming of a new connection, to adapt and maintain communication between cortical regions affected by the disease. Meanwhile, in the cognitive task condition, differences between groups were not as pronounced for both ME and MM tasks. Our findings provide a reference for future studies focusing on group differences in task-related EEG functional connectivity.

### 4.3. Reduction of EEG Connectivity from Rest to Task Condition Due to Cognitive Decline

Previous studies have reported the inter-state change in EEG functional connectivity for AD in comparison with HC. For instance, compared to healthy participants, individuals with AD showed less coherence-based connectivity increase from resting state to visual stimulation state, potentially indicating a failure in task-related functional reorganization in AD brains [107]. Similarly, we observed a significant decrease in theta band PLI-based EEG connectivity from rest to task in individuals with AD, while an overall increase in HC subjects (see Figure 7). In contrast, individuals with MCI showed an increase of theta band PLI-based EEG connectivity in the fronto-central areas in both the MM and ME conditions (see Figure 7). This pattern aligns with the posterior-to-anterior shift in aging (PASA) phenomenon, where earlier studies found that older adults tend to show an increase of frontal activation during cognitively demanding tasks [108,109,110]. According to the PASA hypothesis [109,110], the recruitment of the frontal region reflects a compensatory mechanism, which could also account for the increased fronto-central connectivity observed in individuals with MCI in the current study. However, for individuals with AD, since their cognitive declines are more prominent, the overall connectivity reduction may reflect a general struggle to switch to an optimal functional status during cognitively demanding working memory tasks as a result of degenerated brain networks. Given the limited spatial resolution of EEG, future studies with different neuroimaging modalities such as fMRI or MEG are needed to explore this aspect further.

### 4.4. Practical Use of the Proposed Diagnosis Approach

Compared to other AD/MCI diagnosis-assistive methods, including cerebrospinal fluid (CSF) testing or neuroimaging techniques such as MRI and PET, EEG offers several advantages in terms of its practicality. CSF testing requires invasive procedures that show higher risk for other medical complications. Although MRI or PET are non-invasive or minimally invasive, the procedures require expensive and immobile equipment and specialized clinical infrastructure. In contrast, EEG is non-invasive, cost-effective, and highly portable, making it far more accessible for routine clinical use, follow-up assessments, and even at home monitoring. Importantly, EEG could provide real-time data on brain activity, allowing for the detection of subtle neurophysiological alterations that may not yet be visible in structural scans. This functional sensitivity, especially in early or preclinical stages, makes EEG a valuable tool for timely intervention. With the advancement of computational hardware and further optimization of the analyses, EEG data can be easily digitized and integrated with automated analysis pipelines to provide immediate information for clinical references. In clinical practice, we envision that the current framework can be incorporated into a regular EEG assessment packet, which will include recordings with a 90 s resting session and a 5 min working memory task session. These characteristics—affordability, accessibility, non-invasiveness, and real-time functional insight—position EEG as a highly viable and scalable solution for early and/or ongoing assessment of cognitive decline.

### 4.5. Limitations and Future Works

Although the current study proposed a novel feature extraction framework based on inter-state change of EEG-based connectivity for detecting individuals with MCI, two limitations should be addressed to improve its robustness. First, in the current study, the EEG features of inter-state change were captured using a trial averaging approach, which primarily reflects overall neurophysiological signal shifts. This method does not account for the trial-to-trial dynamics of brain responses throughout the whole session of the working memory task. To enhance the detection of early signs of pathological cognitive decline, it may be beneficial to consider the variation of functional connectivity across time, as this could be more sensitive than averaged signal changes over a long period. In this context, employing a recurrent neural network (RNN) may be more appropriate for capturing the temporal dynamics of the rest-to-task transition in raw EEG signals without performing the feature-level averaging preprocessing. Features automatically extracted by such RNNs could potentially magnify group differences.

Second, sharing a limitation common to many current EEG-based studies, the test-retest reliability of the observed classification performance should be assessed with a multi-session study design, ideally over a span of weeks. Such testing may provide critical insights into the development of a detection system that is sensitive to the pathological course of cognitive decline. In addition, it is crucial to assess the generalizability of WM-TIER across different clinical sites, demographics, and task paradigms. The current study, relying on a single experimental setting, is therefore limited in its capacity to expand the testing for the robustness and reproducibility of the WM-TIER framework’s performance. In particular, the EEG features extracted in WM-TIER may include working memory processes as well as other components such as sustained attention and visual perception. Without the inclusion of other task types (e.g., selective attention task), we cannot test the specific contribution of working memory processing while ruling out the possibility that other cognitive or perceptual processes may also play a role in the detection of cognitive decline. Future research to collect multi-session data with multiple types of cognitive tasks from a demographically diverse dataset will be needed to validate the robustness and generalizability of the proposed framework, ensuring its practical applicability in real-world scenarios for detecting cognitive impairments.

## 5. Conclusions

The current study proposed a novel feature extraction framework that derives the working memory task-induced EEG response for detecting AD/MCI vs. HC. We compared its classification performance to the conventional framework of using only resting or cognitive task EEG. In each framework, we examined three different feature types: relative power, spectral coherence, and filter-bank phase lag index. The results demonstrated that the working memory-based EEG response provides high AD/MCI versus HC classification accuracy, superior to both resting and task EEG features. The dynamic change in EEG from rest to task shows promise as a neural marker for detecting AD/MCI.

## Figures and Tables

**Table 1:** Data Summary of the AD, MCI, and HC groups [Mean (SD)].

Variable	HC	MCI	AD	*p*	Effect Size
	*n* = 27	*n* = 24	*n* = 21		
Gender	17F, 10M	14F, 10M	8F, 13M	0.202	0.211
Age	69.93 (4.98)	70.96 (8.20)	71.19 (5.04)	0.750	0.008
Education (years)	13.44 (3.18)	12.13 (3.76)	11.48 (3.82)	0.153	0.053
MMSE	28.26 (1.79)	26.58 (1.89)	21.62 (6.03)	<0.001	0.379
MoCA	25.89 (3.29)	23.08 (4.11)	16.52 (6.67)	<0.001	0.405

Note. F = female, M = male, HC = healthy controls, MCI = mild cognitive impairment, AD = Alzheimer’s disease, MMSE = mini-mental state examination; MoCA = Montreal cognitive assessment, Gender: chi-square test of independence, Age/education year/MMSE/MoCA: one-way ANOVA.

**Table 2:** Highest LOPO-CV classification performance of AD vs. MCI, AD vs. HC, MCI vs. HC, and AD vs. MCI vs. HC using rsEEG, task EEG (ME-EEG and MM-EEG), and working memory task-induced EEG response (ME-TIER and MM-TIER) with FB-PLI-based features based on different classifiers (LDA, KNN, SVM, and LR).

		LDA		KNN		SVM		LR	
		ACC	d $/N_{c}$	ACC	d $/N_{c}$	ACC	d $/N_{c}$	ACC	d $/N_{c}$
AD vs. MCI	rsEEG	88.89	13/16	84.44	18/20	88.89	11/15	88.89	11/15
	ME-EEG	84.44	44/29	86.67	23/20	84.44	12/13	86.67	25/22
	ME-TIER	95.56	20/21	95.56	9/13	95.56	11/14	95.56	23/21
	MM-EEG	93.33	9/12	84.44	16/18	88.89	23/21	82.22	43/26
	MM-TIER	93.33	16/17	95.56	41/26	97.78	42/26	97.78	41/26
AD vs. HC	rsEEG	89.58	12/16	95.83	15/17	97.92	17/21	89.58	32/25
	ME-EEG	85.42	8/12	83.33	30/27	79.17	2/4	85.42	41/30
	ME-TIER	95.83	21/21	95.83	45/27	95.83	27/22	93.75	14/18
	MM-EEG	83.33	6/7	81.25	15/14	89.58	6/7	81.25	42/28
	MM-TIER	97.92	47/26	95.83	38/25	95.83	34/24	97.92	38/25
MCI vs. HC	rsEEG	86.27	15/18	90.20	47/29	94.12	45/28	90.20	28/26
	ME-EEG	88.24	8/15	94.12	36/27	92.16	49/28	94.12	38/27
	ME-TIER	92.16	11/16	98.04	43/27	98.04	44/27	98.04	41/26
	MM-EEG	94.12	12/16	90.20	10/14	94.12	40/28	90.20	31/28
	MM-TIER	96.08	12/17	98.04	44/27	98.04	21/25	98.04	28/26
AD vs. MCI vs. HC	rsEEG	70.83	10/13	75.00	68/29	75.00	36/24	72.22	37/24
	ME-EEG	66.67	12/15	61.11	68/29	73.61	71/29	72.22	67/29
	ME-TIER	76.39	31/22	83.33	52/27	91.67	65/28	84.72	53/27
	MM-EEG	70.83	22/22	70.83	40/27	80.56	63/30	75.00	52/28
	MM-TIER	79.17	67/29	83.33	71/29	91.67	68/29	93.06	67/29

Note. ACC = accuracy, *d* = the optimal number of features, *N_c_* = number of channels.

## Data Availability

Due to the confidentiality concerns, EEG data in the current study can only be available upon reasonable requests to the first author or the corresponding author.

## Appendix A. Permutation-Based Multiple Comparison Correction for Between-State Analysis

This section addresses the question of whether any theta-band FB-PLI connections significantly increase or decrease within each group (AD, MCI, HC) when transitioning from the resting state to the working memory task. Each subject has a total of 435 FB-PLI functional connectivity values for the theta band per epoch, with 20 epochs for both the resting state and the working memory task. Although the working memory task consists of three different types, we first average the FB-PLI values across task types and perform the below permutation test before further averaging the FB-PLI values across epochs. (Algorithm A1).

**Algorithm A1. Permutation-based correction for multiple between-state comparisons**

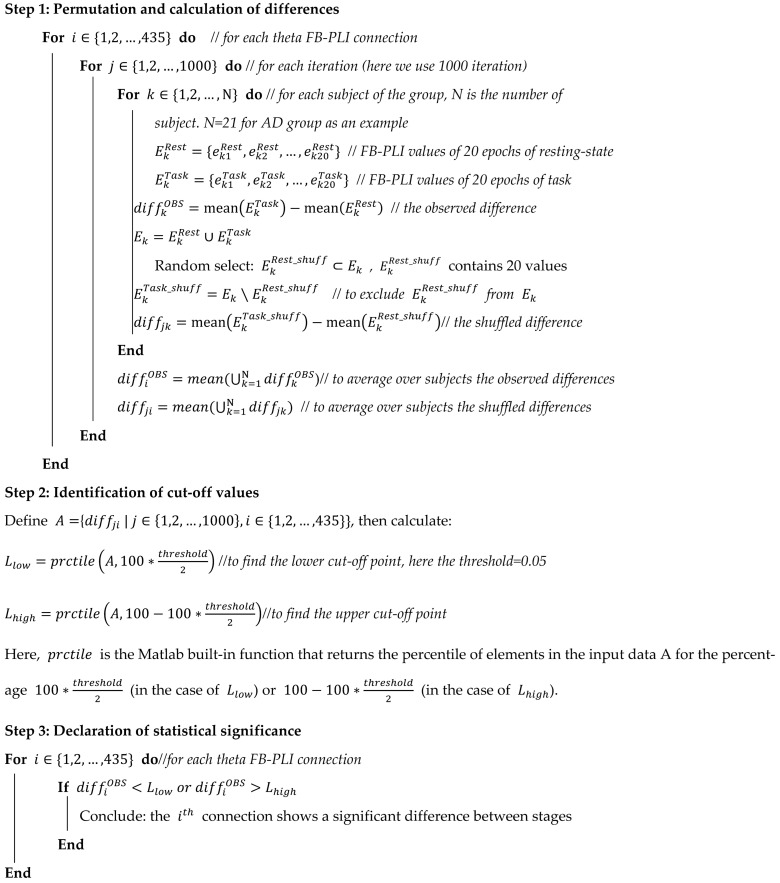

## Appendix B. Permutation-Based Multiple Comparison Correction for Between-Group Analysis

This section addresses the question of whether any theta-band FB-PLI connections significantly increase or decrease in group comparisons (AD vs. MCI, AD vs. HC, MCI vs. HC) for each condition (rest, task, and TIER). (Algorithm A2).

**Algorithm A2. Permutation-based correction for multiple between-group comparisons**
